# Supplementary material for: Orbital-flop transition of superfluid 3He in anisotropic silica aerogel
Source: Nat Commun. 2024 Jan 3;15:201. doi: 10.1038/s41467-023-44557-5 (PMC10764773; doi:10.1038/s41467-023-44557-5)
Supplement: Supplementary file 1 — Supplementary Information [file 41467_2023_44557_MOESM1_ESM.pdf]

SUPPLEMENTARY MATERIAL

The structure factors in Fig. 4 of the main text show $S(\boldsymbol{q})$ out to $q \sim 0.1\ r_0^{-1}$. While the small-angle X-ray scattering data is only dependent upon the small $\boldsymbol{q}$ behavior of $S(\boldsymbol{q})$, we can calculate the full structure factor out to $\boldsymbol{q} = r_0^{-1}$. As seen in Fig. 1, there are oscillations in the intensity at large $\boldsymbol{q}$ arising from the interparticle spacing, showing that the differences in the two anisotropies are still evident at the smallest scale (largest $\boldsymbol{q}$).

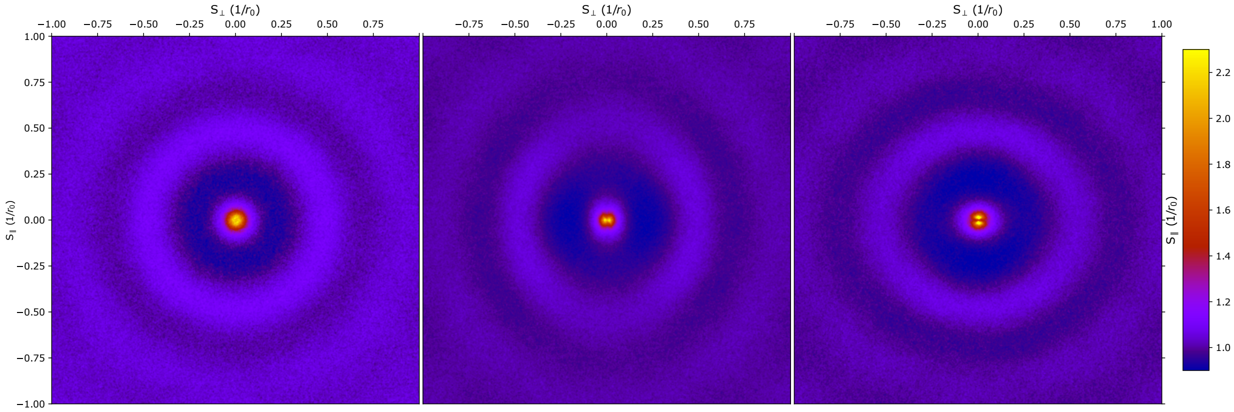

FIG. 1. **The full structure factor.** Isotropic (left), nematic $\epsilon = 0.125$ (center), and planar $\epsilon = 8$ (right) aerogels, as in Fig.4 of the main text, but extended to large q.

The crossover from large to small scale structure (from dipolar to ellipsoidal scattering pattern) can be seen more explicitly by comparing two different slices from the SAXS data. For compressed aerogel, the direction with more intense scattering switches from perpendicular to ϵ (90°) at small q to parallel to ϵ (0°) at larger q as seen in Fig. 2. The length scale at which this crossover occurs is ~ 55 nm (dashed line in Fig. 2), which is consistent with $\xi(T_x, P)$.

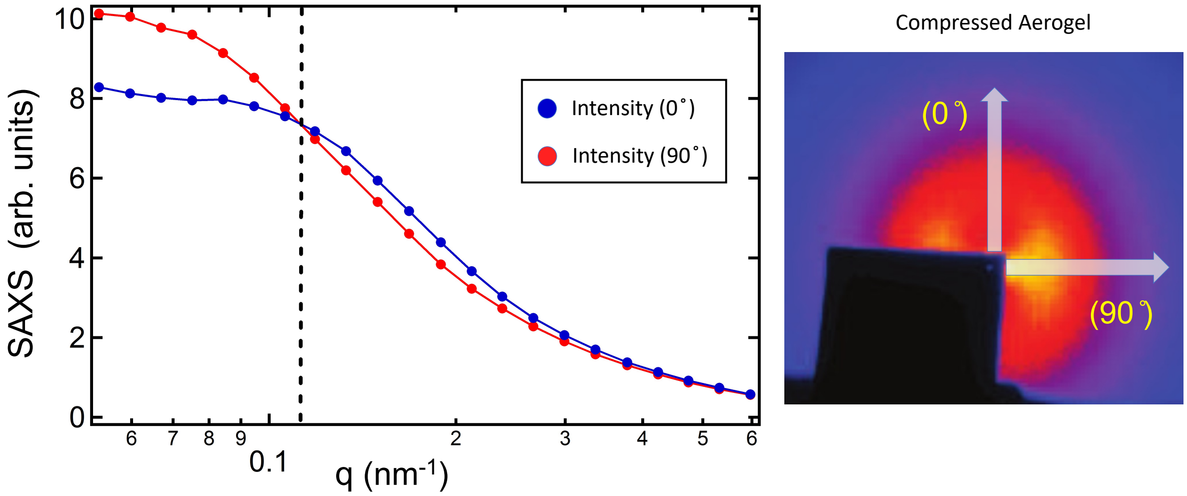

FIG. 2. **Crossover length**. SAXS data of compressed aerogel, same as panel **e** from Fig. 4 of the main text. Blue curve is a slice of the intensity parallel to ϵ while the red curve is a slice perpendicular to ϵ.
